# Atlas VPM: two decades informing on unwarranted variations in health care in Spain

**DOI:** 10.1007/s43999-022-00005-3

**Published:** 2022-07-20

**Authors:** Ester Angulo-Pueyo, Micaela Comendeiro-Maaløe, Francisco Estupiñán-Romero, Natalia Martínez-Lizaga, Manuel Ridao-López, Javier González-Galindo, Ramón Launa-Garcés, Miriam Seral-Rodríguez, Enrique Bernal-Delgado, on behalf of Atlas VPM group, J. A. Goicoechea Salazar, V. D. Cantó Casasola, I. Falcón Alloza, M. D. Muñoyerro Muñiz, M. J. Margolles Martins, H. Sánchez Janáriz, S. Trujillo Alemán, R. Tristancho Ajamil, G. Suarez Rodríguez, M. Estupiñán Ramirez, G. Romero Ruiz, M. M. Navarro Córdoba, L. Muñoz Ortiz, M. Espallargues, G. Oliva, R. Monsalve Torrón, M. C. Pacheco Martínez, F. Pastrana Ara, M. J. Pérez Boillos, C. García Colmenero, C. Verde López, R. Vázquez Mourelle, R. Sanguino, M. C. Castelao, P. Vacas, E. J. Castaño Riera, Y. Muñoz Alonso, M. Zaforteza Dezcallar, E. Carandell Jäger, A. Pujol Buades, D. Medina iBombardó, P. Sáenz Ortiz, A. Cestafé, F. Riera Sanz, J. C. Oliva Pérez, R. Celada, F. L. Sánchez Prieto, J. Palomar Rodríguez, R. García, J. Gorricho, M. Iragui, B. Ibáñez-Beroiz, J. Librero-López, E. Millán Ortuondo, I. Garmendia Navarro, T. Goretty Escobar, C. Jiménez, J. Calabuig, R. Sotoca, S. Peiró Moreno, I. Hurtado

**Affiliations:** Institute for Health Sciences in Aragon (IACS), Aragon 50009 Zaragoza, Spain

**Keywords:** Variations in health care, Hospital care, Primary care, Small-area analysis, Hierarchical analysis, Time-series analysis

## Abstract

Since the early 2000’s, the Atlas of Variations in Medical Practice in the Spanish National Health System (namely, Atlas VPM) has been analysing and informing unwarranted variations in health care provision and outcomes in the Spanish Health System.

Atlas VPM covers a two-fold perspective: a geographic one, where unwarranted variations would reflect the uneven exposure of the population to health care as a consequence of the place of residence; and, a provider-specific approach, where unwarranted variations would reflect differences in utilisation and outcomes that are at provider-level.

Building on routine data (hospital and primary care electronic records, administrative data, geographic information, etc.) Atlas VPM has adapted the classical small area methods and has included a large panoply of techniques, such as Bayesian methods, hierarchical modelling or time-series forecasting.

Led by the Data Science for Health Services and Policy Research group at the Institute for Health Sciences in Aragon, Atlas VPM implies a linkage and exchange process with the 17 Departments of Health of the Spanish regions where the research agenda is shared and research outcomes are translated into profiling and benchmarking interactive tools meant to facilitate clinical and policy decision-making.

## Context and purpose

The “Atlas of Variations in Medical Practice in the Spanish National Health System -Atlas VPM- [1] was conceived in the early 2000’s, getting inspiration from the Dartmouth Atlas of Healthcare theories and epistemic approach.

Atlas VPM was primarily thought as a tool to raise awareness on the magnitude, ubiquity, and consequences of unwarranted variations in health care, with a view to foster a debate among decision makers, particularly clinicians, managers and policy makers at local, regional and national level.

Accordingly, the research agenda, the outputs and the messages reflect the institutional design of the Spanish health system. Noticeably, Atlas VPM inception coincided with the completion of the health system devolution to the regions (so namely, autonomous communities) where the responsibilities for financing, planning and services purchasing reside. Within the region, virtually all the residents are registered to a primary care doctor and administratively allocated to small primary care areas. A group of primary care areas composes the, so called, health care areas, administrative circumscriptions where hospital and outpatient specialised care is provided. So, from a decision-making standpoint this institutional design translates into different decision instances (the region, the health care area, the hospital and the primary-care centre) that represent the main units of analysis of Atlas VPM.

Moreover, with a view to provide more actionable policy messages, Atlas VPM classifies unwarranted variations in five categories; thus: a) underuse of highly effective services; b) provision of highly effective services in non-eligible populations; c) provision of highly effective services when a more cost-effective services is accessible; d) provision of essentially ineffective services; and e) provision of low-quality or unsafe care.

## Governance

At the beginning, Atlas VPM was conceived as a multisite research project. So did it happen when, in 2001, it was granted with a national grant from the national research council. However, Atlas VPM translational nature and the possibility of being a tool for the transformation of the Spanish National Health System (SNHS), in 2003 the project pioneered a linkage and exchange process with the different departments of health of the Spanish regions (i.e., equivalent to federal states when it comes to health and healthcare provision). This process finalised with the creation of a governance body, composed of the Atlas VPM researchers, representatives of the 17 Spanish regions (namely, Autonomous Communities) and the Data Science for Health Services and Policy Research group at the Institute for Health Sciences in Aragon (IACS), Atlas VPM founders and leaders of the project.

The governing body plays a two-fold role; on the one hand, it agrees on a research agenda that is of the interest for the regional health departments; and, on the other hand, acts as the warrantor for the proper use of the data provided to the leading group, so that, is in compliance with the legal provisions on the reuse and pooling of individual administrative and clinical data.

In terms of financing, Atlas VPM is mainly funded via competitive public grants (around 81%). Nonetheless, over the years, some regional health departments commissioned specific Atlases and Reports aiming to inform some of their policies, e.g. variation in the implementation of the strategy on stroke, gender inequalities in potentially avoidable hospitalisation, variations in chronic care with specific focus on socioeconomic and gender inequalities, or variations in low-value care, etc. Finally, a small amount of financing comes from private firms that contract advisory services based on the Atlases outputs.

## Methods and data

Atlas VPM seeks to describe and analyse unwarranted variations in health care from a two-fold perspective; thus, a) a geographic perspective, where unwarranted variations reflect the uneven exposure of the resident population to health care; and, b) a provider-specific approach, where unwarranted variations represent the different outcomes that patients would have if they received care in a different provider.

### Units of analysis

Atlas VPM shows variation across geographical areas and hospitals or primary care providers. In the former, the cases of interest are allocated to the place of residence of the individuals while in the provider-specific approach the cases of interest are allocated to the place where the patients were treated, either hospital or primary care setting.

Interestingly, in the geographical approach, the decision on what areas result meaningful for the purposes of the analyses is delimited by the institutional design of the Spanish Health System. All the Spanish population is allocated to administrative areas, rather stable over time. Thus, the Spanish residents are registered to a primary care physician that works in primary care settings. These primary care settings are based in administrative areas that, in turn, compose the, so called, health care areas, where hospital and outpatients specialised care is provided. In the case of tertiary services, different health care areas compose a grand health care area. So, when it comes to the geographic perspective, depending on the topic of interest, the units of analysis are primary care areas (e.g. diabetes care), health care areas (e.g. knee replacement) or grand areas (e.g. CABG) [2].

### Data

Atlas VPM reuses routine data mainly from electronic records from hospital admissions and primary care visits for the identification and selection of cases (e.g. surgical procedures of interest, specific diseases, quality and safety events), the analysis of clinical attributes of the patient (e.g. comorbidities, concurrent surgery), the analysis of administrative features worth to collect (e.g. admission and discharge data, date of surgery) and for the identification of the place of residence (i.e. primary, health care or grand area where the patient resides). In addition, Atlas VPM maintains a geographical information system (e.g., mapping vectors) that is interoperable with any other data source including geographical information of any kind. Currently, Atlas VPM has started the exploitation of primary care prescription records and pharmaceutical claims to investigate pharmaceutical care in diabetic patients and to assess unwarranted variations in the prescription of pain-killers and antibiotics.

In addition, Atlas VPM has reused administrative data from health and economic surveys to proxy the socioeconomic level of the geographic area where the individual resides [3] and, more recently, the insurance records where levels of copayment are registered for each individual, to proxy the wealth of the individual receiving primary care [4].

Lastly, Atlas VPM uses supply data at both, area and hospital levels, so to stratify the analyses according to specific supply features and improve comparisons across providers (i.e. hospitals with similar services, areas served by similar providers).

### Data management

Atlases in Atlas VPM are based on data pooling of anonymised individual data, as well as on aggregated level data provided by the Spanish regional healthcare systems. Data collections are stored and maintained in a secured environment in IACS premises where just Atlas VPM researchers based in IACS have logged access.

The regional representatives of the Atlas VPM, once data sources at origin are consolidated, send the universe of hospitals’ admissions duly anonymised (approximately 5 to 6 million discharges per year) or subsets of aggregated information in the case of primary care visits according to a specific data model. Both subsets contain information on the area of residence and co-payment level.

**Fig. 1:**
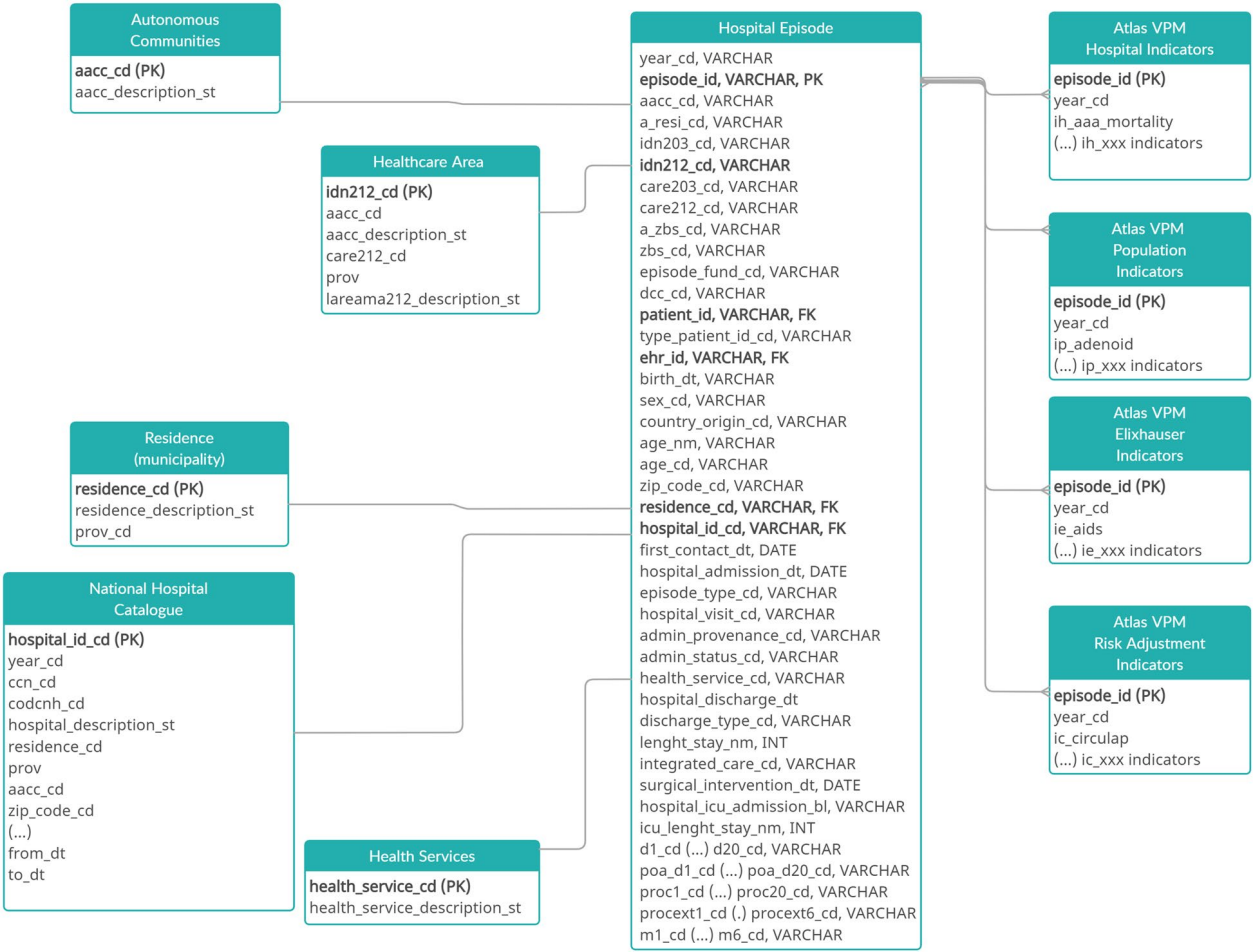
Entity-relationship diagram of the Atlas VPM database

Once received, Atlas VPM data managers test the data received and run transformation rules to comply with the common data model for the project, thus to be interoperable (common data model available at https://doi.org/10.5281/zenodo.6006136, Fig. 1).

In the particular case of hospital admission, once the transformation process is over, the data managers run several algorithms that flag events of interest (e.g. whether the patient has a specific surgery, whether there is a quality event, whether the patient endures some comorbidities or whether the health provider offers a specific service, for example, neonatal intensive care). Finally, each admission is allocated to the area of residence, primary, healthcare or grand areas and to the specific provider where the patient was treated.

Should be noted that, in the geographic perspective, Atlas VPM has implemented an interoperable solution for the geolocation of the units of analyses (geographical frontiers of the care areas and care providers) compliant with INSPIRE [5].

## Analyses

A panoply of analytical techniques is used to respond to the Atlas VPM questions. Broadly speaking, for geographical perspective Atlas VPM builds on small area analyses and disease mapping techniques, while in the provider-specific perspective, it follows hierarchical analysis, both cross-sectional and time-series.

### Small area analyses

Besides the use of age and sex standardised rates (direct methods) and standardised utilisation ratio (indirect method), an adaptation of the classical statistics of variation was implemented [6] to cope with the extra-heterogeneity intrinsic to the small and uneven size of the areas in Spain, particularly when working with the rather small primary care areas. So, two Bayesian techniques are being regularly used: a) the Besag-York-Mollié approach to define how likely a primary care area is above (or below) the expected; and, b) Shared-Component Analysis to estimate the likelihood of getting care or enduring a quality event for a man (as compared to a woman) or vice versa; or, likewise, for older people compared to younger patients [7–9].

### Provider-specific analyses

Atlas VPM uses various approaches in the analyses of variation at provider level, notably, hierarchical methods and forecasting methods.

Hierarchical methods are used to assess which fraction of the variance can be explained by differences in providers performance beyond patients’ differences. These techniques also allow the assessment of random effects at hospital level, thus analysing if hospitals perform differently when treating some specific patients’ subgroups [10].

Forecasting methods allows predicting 24-months hospitals’ performance for a variety of quality indicators; specifically, Atlas VPM is using the Holt-Winters exponential smoothing forecasting method which allows differentiating level, trend and seasonality [11].

## Outcomes and reporting tools

Atlas VPM analyses systematically unwarranted variations throughout a number indicators covering utilisation, access, equity, quality and safety usually grouped thematic areas policy relevant. Up to the moment of writing this work, Atlas VPM has addressed twelve different topics (Table 1). The first Atlas, Atlas on Orthopaedic and Trauma Surgery [12], was published in 2005; since then, more than 20 Atlases have been launched.

**Table 1:** Atlases’ topics and year of the first publications and following updates

Domain	1st issue (year)	Update (years)
Orthopaedic and Trauma Surgery	2005	2014,2017, 2020
General Surgery	2005	–
Paediatric Hospitalisations	2006	2021
Hospitalisations for Cardiovascular Conditions	2007	2014
Hospitalisations for Mental Health Conditions	2008	–
Oncologic surgery	2009	2021
Elderly Hospitalisations	2010	–
Potentially Avoidable Hospitalisations	2011	2015,2017, 2019
Ischemic Cerebrovascular Disease Management	2013	2014
Lower Value Care	2017	2020
Quality of hospital care	2021	2021
Diabetes care	2017	2022

In addition to the Atlases, Atlas VPM also prepares tailored reports to regional authorities interested in the evaluation of their health care policies. So for example, Atlas VPM elaborated reports for the Basque Country and Andalusia about potentially avoidable hospitalisations and low value care respectively, for the Canary Islands about orthopaedics and lower value care and for the Balearic Islands on stroke care. Recently, Atlas VPM has implemented the first Atlas of Healthcare for the autonomous community of Cantabria.

### Reporting tools

Atlases were initially published as paper editions [13]. Since 2015, Atlas VPM has turned online facilitating interactive online tools for users to navigate maps, time-series charts, forecast and performance profiles.

### Maps

**Fig. 2:**
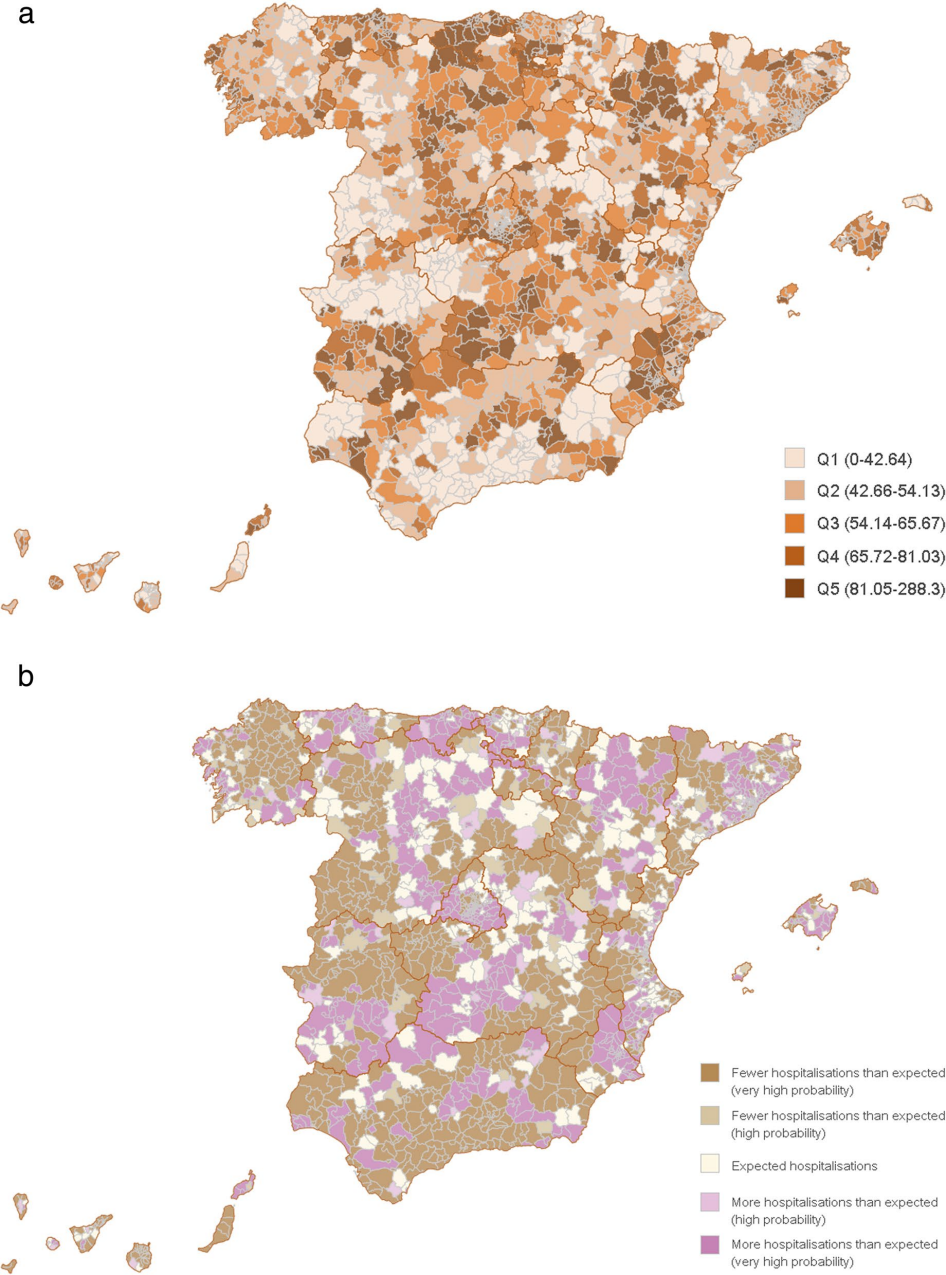
Potentially avoidable hospitalisations in primary care areas **a** age-sex standardised rates per 10,000 inhabitants. The darker the brown colour the higher the rate of avoidable hospitalisations. Primary care areas are clustered into 5 quintiles according to their rate value (Q1 to Q5). Legend indicates the range of standardised rates values within each quintile; **b** probability for a standardised ratio to be above or below the expected as estimated by Besag-York-Mollié. Legend indicates how probable for a primary care area would be to endure more (or less) admissions than those expected. More details in https://www.atlasvpm.org/wp-content/uploads/2019/10/Metodologi%CC%81a-y-co%CC%81digos-Atlas-HPE-ZBS-Abril-2019.pdf) (extracted from https://www.atlasvpm.org/atlas/hpe-zbs/ [14])

In the geographic approach, maps will represent the traditional standardised rates or standardised ratios (either using the indirect method or Bayesian approach) reported at health care or primary care areas, depending on the research question (Fig. 2). As an example of this approach, Fig. 2a showed the standardised rates of potentially avoidable hospitalisations in the primary care areas in Spain while Fig. 2b shows the probability for the standardised utilisation ratio -estimated using the Besag-York-Mollie method – to statistically exceed the value of 1 (greater risk of PAH than expected) or to be statistically lower than 1 (lesser risk of PAH than expected).

When appropriate, maps are used to report on shared-component estimates, for example, the probability for a ratio to be true, or the likelihood for women (as compared to men) or vice versa to get a particular procedure or experiment a specific event (likewise when comparing age groups of interest) [15].

**Fig. 3:**
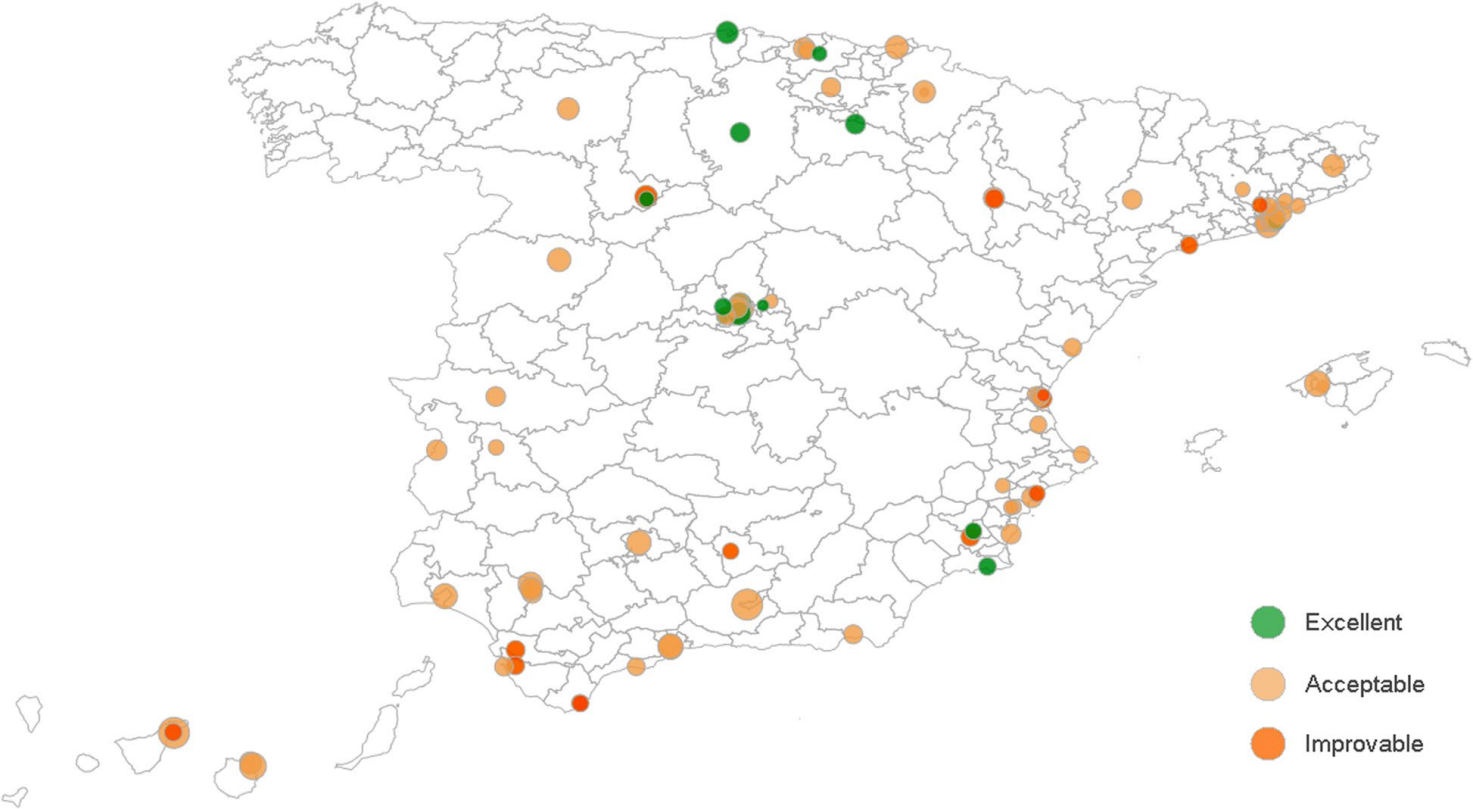
Acute myocardial infarct mortality in hospitals treating high complex patients in Spain 2018. Each circle represents a hospital, the size of the circle indicates the volume of patients at risk at each hospital, whereas colour denotes their performance level, being green excellent, light orange acceptable and dark orange need for improvement (extracted from https://www.atlasvpm.org/atlas/calidad-2018/ [16])

In the provider-specific approach, maps serve as the basis for hospitals geolocation but adjusted risks are represented as circles whose size represents the number of patients at risk and colour denotes different risks of the event of interest, and whether they are statistically significant (Fig. 3).

### Time series charts and forecasts

**Fig. 4:**
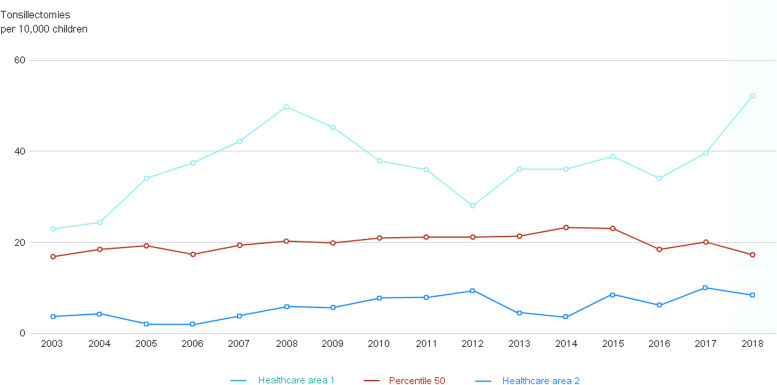
Standardised rates of tonsillectomy per 10,000 children below 15 years old from 2003 to 2018 in two health care areas in Spain. The red line represents the rates at percentile 50 for all the healthcare areas over the study period (extracted from https://www.atlasvpm.org/atlas/pediatria-2018/ [17]

Irrespective of the approach, whether geographic or provider-specific, all the atlases report on the evolution of performance indicators since the first available data. Time series are usually reported annually and values are compared to the median evolution of each indicator at the different units of analysis (Fig. 4). For example, Fig. 4 shows the evolution of tonsillectomy rate in two healthcare areas from 2003 to 2018 compared to the rates in percentile 50 (P50). In this case healthcare area 1 showed rates above P50 consistently over the analysed period, whereas in healthcare area 2 tonsillectomy rates were consistently below P50.

**Fig. 5:**
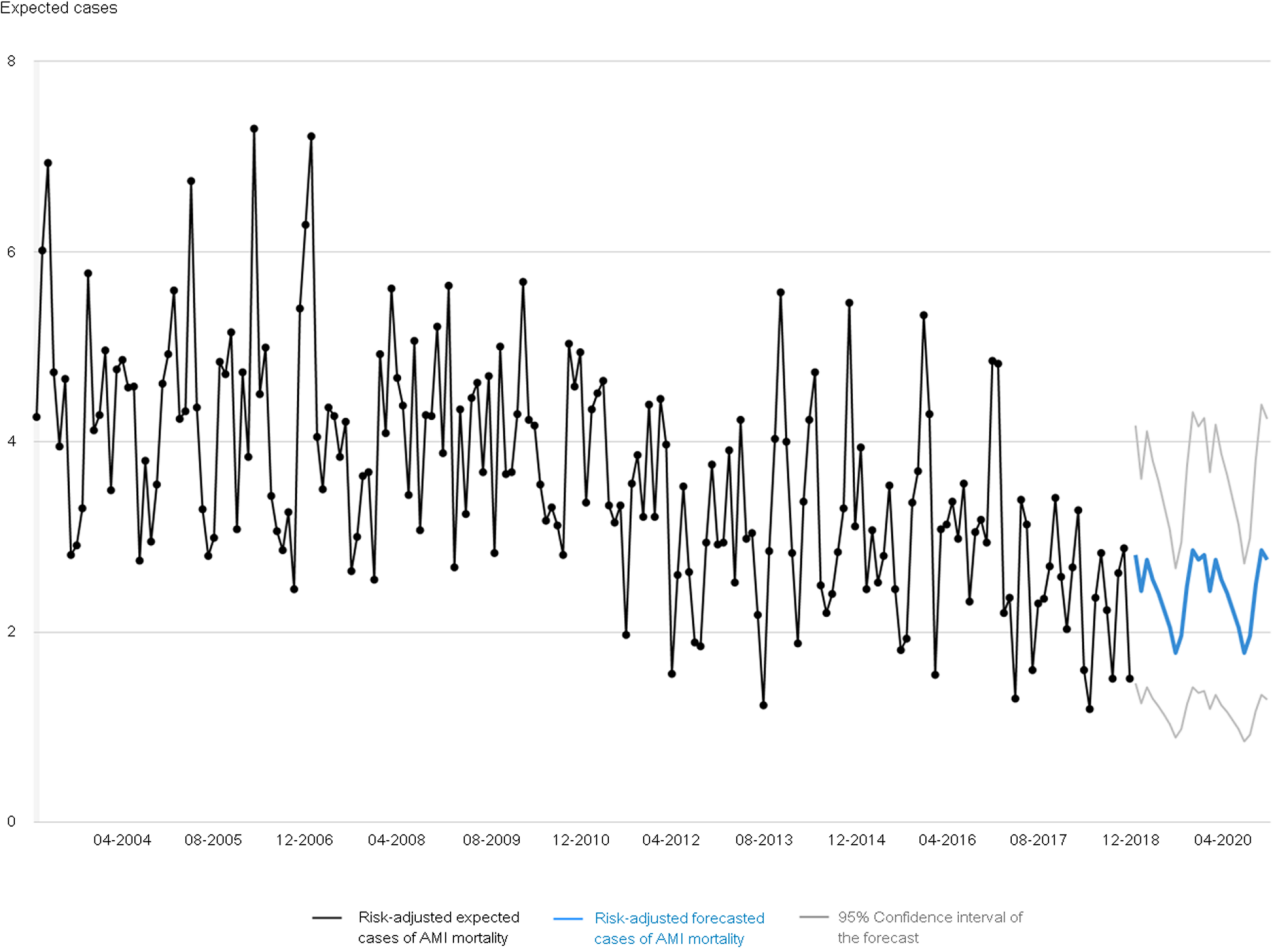
Monthly acute myocardial infarction (AMI) mortality (risk-adjusted expected cases) in a Spanish hospital treating high complex patients from 2003 to 2018 (black line). Blue line represents the forecast of the performance of the hospital in the next 24 months with the grey lines depicting the confidence interval (extracted from https://www.atlasvpm.org/atlas/calidad-2018/ [16])

In addition, in those Atlases reporting hospital quality, time series are reported at a monthly-basis together with a 24-month forecast for each hospital based on its behaviour since first available data (Fig. 5) using the Holt-Winters exponential smoothing forecasting method [11].

### Profiling and benchmarking

All the Atlases provide a performance profile of the units of analyses. Thus, all the performance indicators for each healthcare area, primary care area or hospital are represented together and compared with a benchmark.

**Fig. 6:**
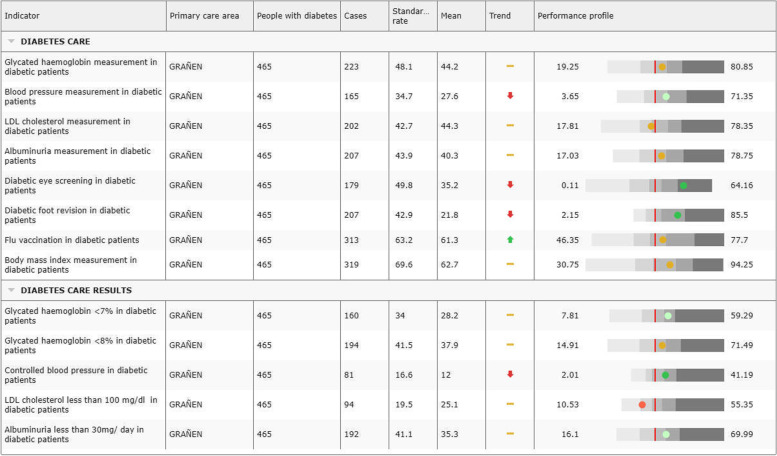
Primary care area profile showing the diabetes care provided by a specific primary care centre and its results. Coloured circles represent the level of performance of the specific area as compared to the regional mean: green circles denote better performance, the orange ones indicate same performance and the red ones provide an indication of the need for improvement. Arrows represent the trend of the indicator compared with the previous year, meaning increase, decrease or no change interval (extracted from https://www.atlasvpm.org/atlasvpm/diabetes/atlas.html [4]

In geographic analyses, the benchmark represents the distance (i.e. statistical difference) to a specific percentile of the distribution of values in all the areas of comparison; usually percentile 50, although when a theory exists on *“less is better”* the comparison uses percentile 25. When it comes to provider-specific analyses, the benchmark represents the distance between the unit of interest and the average rate as estimated from the hierarchical models. In the graphic representation used in the Atlas, the circle represent the actual value for a specific performance indicator, and the colour, how different from the benchmarking is performing. As an example, Fig. 6 shows a primary care area profile for different care indicators as compared to the regional mean which act as a benchmark (Fig. 6).

## Atlas VPM dissemination tools

Besides its website that contains the digital Atlases tools (https://www.atlasvpm.org/atlas/), Atlas VPM sought a multifaceted approach to disseminate and teach the Atlases concepts, methods and results among decision makers and academia.

As for decision-makers, Atlas VPM participates in national or regional *fora* where decision makers usually meet and in academic events where decision makers get training. In addition, Atlas VPM has used health care champions and opinion leaders as ambassadors of the Atlas VPM’s achievements and potential.

As for academia, Atlas VPM is a leading partner in several research networks at national level and contributes to the major scientific events in the country within the domains of Chronic Care, Public Health, Health Administration and, Health Economics and Health Policy.

## Outstanding achievements

### Atlas VPM recognised as initiative of interest for the Spanish Health System

Atlas VPM methods and outputs were audited at national level as part of an accreditation process launched by the Ministry of Health seeking the identification of data-driven initiatives aiming at the evaluation of health care policies. Atlas VPM earned recognition as “Initiative of Interest” for the Spanish Health System (https://www.sanidad.gob.es/estadEstudios/estadisticas/sisInfSanSNS/registros/docs/Atlas_VPM.pdf).

### Atlas VPM went international

Soon in 2009, Atlas VPM designed a demonstration project whose purpose was replicating the principles and methodologies developed in Spain in the analysis of unwarranted variations, in a number of European Health Systems, with different institutional design. So, the so-called ECHO project analysed the health care provided to virtually all the population in England, Denmark, Portugal, Slovenia and Spain (http://echo-health.eu/).

The achievements in ECHO have served to inform some initiatives by the European Commision (Health Systems Performance Assessment group) and OECD (Health care quality indicators project) as well as some strategic debates at EU level on the development of research infrastructures for the transformation of health systems (JA INFACT https://www.inf-act.eu/).

Atlas VPM has joined international initiatives as the Wennberg International Collaborative (https://wennbergcollaborative.org/), as part of foundational partners, and ICCONIC (https://hnhccomparisoncollaborative.wordpress.com/) where new developments on the analyses of within- and cross-countries variation are carried out.

The TEHDAS Joint Action (https://tehdas.eu) has selected Atlas VPM among the initiatives of interest for the development of the European Health Data Space for secondary use of routine data. A particular interest has been paid to the Atlas VPM proceedings on semantic interoperability and the data quality assurance system implemented within the project's data management plan.

## Conclusions

Atlas VPM has demonstrated to be an outstanding research initiative whose success has built on some foundations; so, 1) although incepted as a research initiative, a broad community of health policy research and potential end users have supported and co-created the project from the beginning; 2) as an immediate consequence, the research agenda, linked to the policy priorities, is shared and agreed with relevant actors in the regions; 3) with a view to be meaningful, the research questions are well rooted in the institutional design of the health system; 4) the methodologies are sound and in constant revision and improvement; 5) the research outputs are thought and reported to foster policy action; and, 6) the research outputs are made public and open to all the interested parties, from the policy-makers to the citizens.
